# Return of the Living Dead Gut – A Case Report of Ischemic Colitis Identified on Point of Care Ultrasound 

**DOI:** 10.24908/pocus.v9i1.16950

**Published:** 2024-04-22

**Authors:** Kandria Ledesma, Joseph Kim, Allison Cohen, Nicholas Bielawa, Mathew Nelson

**Affiliations:** 1 North Shore University Hospital Manhasset, NY USA; 2 Donald and Barbara Zucker School of Medicine at Hofstra and North Shore University Hospital Manhasset, NY USA

**Keywords:** Emergency Medicine, Ischemic Colitis, Bowel Ultrasound, Case Report, Abdominal point of care ultrasound

## Abstract

Ischemic colitis is the most common form of gastrointestinal ischemia 1. The diagnosis of ischemic colitis is made by clinical data and computed tomography (CT) imaging of the abdomen and pelvis 1. While colonoscopy is considered the gold standard for diagnosis, this is not performed in the emergency department (ED) 2. Few studies have been performed to describe the sonographic findings of ischemic colitis using point of care ultrasound (POCUS). We report a case that highlights the sonographic findings of ischemic colitis in a patient who had two separate visits to the ED, showcasing the utility of POCUS in making this diagnosis. POCUS can be used as a diagnostic tool for early detection of ischemic colitis leading to prompt treatment with antibiotics, CT imaging, and surgical consultation.

## Introduction

Ischemic colitis is one of the most common causes of gastrointestinal ischemia. This disorder has two major types: gangrenous and non-gangrenous, with gangrenous being the most severe given higher associated morbidity and mortality 3. Non-gangrenous ischemic colitis is more common, representing approximately 80-85% of cases of ischemic colitis and is transient and self-limiting. Non-gangrenous ischemic colitis is often caused by acute episodes of hypoperfusion, generally secondary to a low-flow state 4.

The incidence of ischemic colitis is often underestimated due it its vague presenting symptoms, including abdominal pain, rectal bleeding, diarrhea, nausea, and vomiting 5. Although it can occur at any age, ischemic colitis increases with age, especially after age 49-years. Risk factors include hypertension, diabetes, coronary artery disease, dyslipidemia, chronic obstructive pulmonary disease, acute hypotension, and atrial fibrillation 6. In the emergency department (ED) these patients often require a computed tomography (CT) scan of the abdomen and pelvis to make the diagnosis; however, this can lead to a delay in making the diagnosis. Abdominal POCUS is increasingly performed in the ED for patients presenting with acute abdominal pain and can potentially help aide in making the difficult diagnosis of ischemic colitis. The sonographic findings of ischemic colitis include symmetric bowel wall thickening greater than 3mm, segmental (greater than 10 cm) colonic involvement, hyperechoic pericolonic fat enhancement, decreased or absence of bowel wall Doppler flow, pneumatosis, and/or the presence of free fluid 3, 7, 8, 9, 10. In the ED abdominal POCUS is often performed using a curvilinear transducer due to the depth of the bowel and the large area that needs to be evaluated.

We report a case that highlights the progression of sonographic findings of ischemic colitis in a patient who had two separate ED visits for abdominal pain.

## Case Report

**Figure 1:**
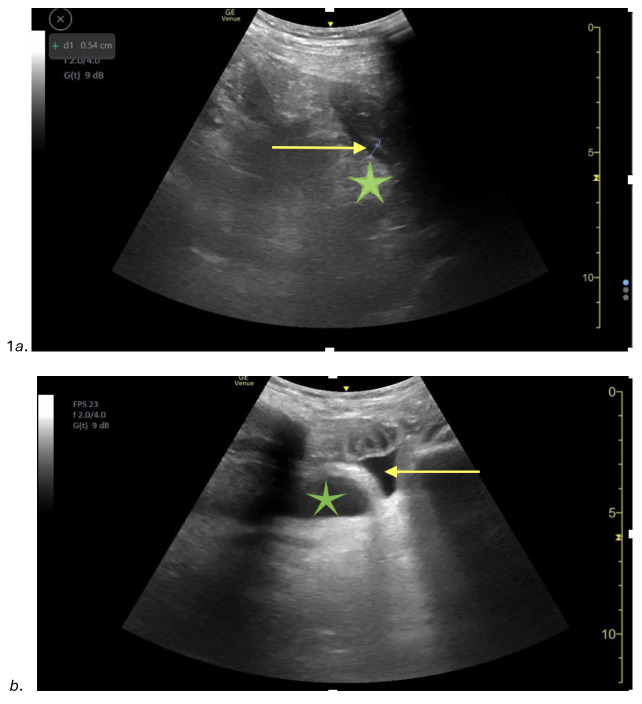
(a): A point of care ultrasound (POCUS) image of the lower abdomen using a curvilinear probe showing a thickened and edematous bowel wall (arrow) and enhancement of the pericolonic fat (star). (b): A point of care ultrasound (POCUS) image of the lower abdomen showing a dilated loop of bowel by the bladder (star) with evidence of free-fluid (arrow) and enhancement of pericolonic fat.

An 82-year-old female with a past medical history of aortic insufficiency and prior surgical history of a right incarcerated femoral hernia repair and incisional hernia repair with mesh placement presented to the ED with abdominal pain, vomiting, and non-bloody diarrhea for the last couple of days. The patient reported diffuse constant abdominal pain, which was worse in the lower abdomen, and decreased oral intake. Upon arrival to the ED, the patient’s blood pressure was 156/81mm Hg, heart rate was 156 beats per minute, respiratory rate was 24 breaths per minute, and temperature was 37.5 degrees Celsius (99.5 Degrees Fahrenheit). On physical exam, the patient was well appearing, tachycardic with an irregular heart rate. The patient’s abdomen was soft, diffusely tender to palpation, with normal bowel sounds and no peritoneal signs. Abdominal POCUS was performed, which showed bowel wall thickening of 0.54 cm (Figure 1a) and free fluid surrounding an area of thickened bowel with enhancement of pericolonic fat (Figure 1b). 

Laboratory findings were significant for an elevated white blood cell count (WBC) of 15.39 K/uL, an international normalized ratio (INR) of 3.14, and a venous blood gas (VBG) lactate of 1.9 mmol/L. A CT Angiography (CTA) of the abdomen and pelvis was ordered for concern for bowel ischemia versus colitis, in the setting of atrial fibrillation, and abdominal pain. The CTA demonstrated a thickened transverse colon wall concerning for colitis, small volume ascites in the pelvis, and no evidence of mesenteric arterial stenosis. The patient received a dose of piperacillin/tazobactam, intravenous fluids, and morphine. Surgery was consulted and the patient was advised to be admitted to the hospital for antibiotics and possible surgical intervention. However, symptoms improved with pain medication, fluids, and antibiotics, and the patient decided to leave against medical advice.

**Figure 2:**
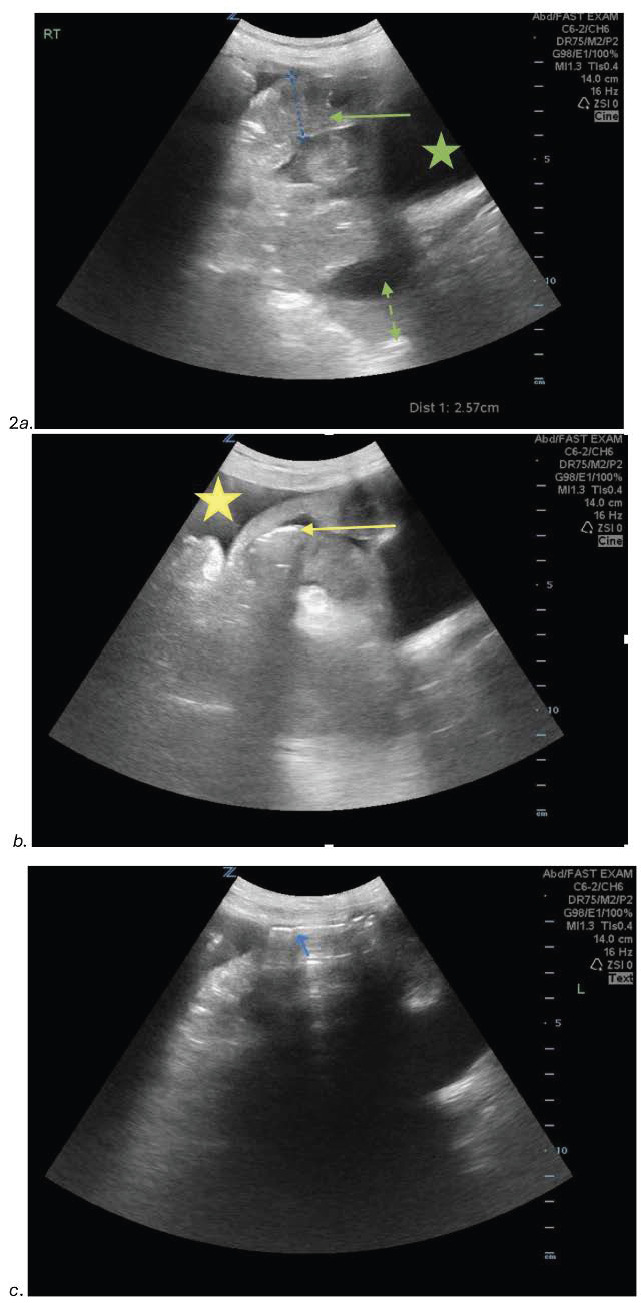
(a): A point of care ultrasound (POCUS) image of the abdomen with a curvilinear probe showing a dilated loop of small bowel (arrow) with free fluid (dashed arrow), and a visualization of the bladder (star). (b): A point of care ultrasound (POCUS) image of the abdomen showing dilated loops of small bowel with evidence of hyperechoic foci within the bowel wall (arrow) and the presence of free intra-abdominal fluid (star). (c): A point of care ultrasound (POCUS) image of the right upper quadrant in sagittal orientation showing a-lines within the abdomen (arrow) with reverberation artifact.

Two days later, the patient returned with worsening abdominal pain, diarrhea, and vomiting. Her vital signs were within normal limits and her abdominal exam was unchanged. A repeat POCUS was performed which revealed free intraabdominal fluid, dilated loops of small bowel (Figure 2a, b), evidence of hyperechoic foci within the bowel wall (Figure 2b), and a-lines within the abdomen concerning for pneumatosis (Figure 2c). 

A repeat CTA of the abdomen and pelvis showed long segment bowel colitis with associated hypo-enhancement concerning for bowel ischemia. Significant laboratory findings were a WBC of 17.60 K/uL, an INR of 4.02, and a VBG lactate of 3.9 mmol/L. The patient was admitted to the surgical service and taken emergently to the operating room (OR). Operative reports indicated a long segment of ischemic small bowel requiring resection, with associated mesenteric edema and ascites, and required a bowel resection for ischemic necrosis of the small bowel. The patient was then taken to the surgical intensive care unit. The patient was taken back to the OR at a later time for repeat evaluation showing poor perfusion of the terminal ileum requiring an ileocecectomy. The patient improved after being monitored in the intensive care unit and was transferred to the floor. Her course was complicated by a urinary tract infection. She remained in the hospital for 16 days and was discharged to a rehab center.

## Discussion

Abdominal pain is one of the most common presenting symptoms in the ED 11. In the evaluation of a patient presenting with abdominal pain, emergency physicians must keep the diagnosis of ischemic colitis in mind. It is a challenging diagnosis to make due to its wide spectrum of clinical symptoms and non-specific clinical, laboratory, and imaging findings 5. The differential for patients with these presenting symptoms of pain, nausea, vomiting, and diarrhea is broad, so ischemic bowel can be overlooked due to more common pathologies. Often, patients undergo CT imaging of the abdomen and pelvis to help elucidate the cause of their abdominal pain; however, as was in the case of our patient, findings can be non-specific. Additionally, CT imaging is associated with higher cost, increased length of stay, and ionizing radiation.

Abdominal POCUS in the ED has become an invaluable tool to help guide management of patients presenting with abdominal pain. More recently, the use of POCUS has expanded to the evaluation of bowel pathologies including small bowel obstruction, diverticulitis, and colitis 12, 13, 14, 15. Previous studies have shown that ultrasound has a sensitivity of 95% and a positive predictive value of 87.5% when evaluating for ischemic colitis 3, 10. One study found that altered pericolic fat and pancolitis on ultrasonography predicted a more severe case of ischemic colitis 7; another study found that the absence of arterial flow in the wall of an ischemic colon is more associated with an unfavorable outcome than early clinical and laboratory findings with a sensitivity and specificity of 82% and 92% 9. Doppler images were not available for our case. The use of POCUS in evaluating bowel pathology is expanding. It is often performed with either a low frequency curvilinear transducer or a high frequency linear transducer depending on the patient’s habitus or depth of the structure of interest. Image acquisition often begins at the point of maximal tenderness on the patient’s abdomen. The bowel is then scanned using a lawn mower technique to ensure that the entire bowel is visualized. Bowel wall thickness measurement requires accurate identification of the mucosa-lumen interface and serosal interface. The thickness measurements are best taken with a linear probe that has better resolution compared to a curved array probe. For this patient, a curvilinear transducer was chosen due to the depth of the bowel. A limitation of using the curvilinear transducer is decreased resolution of the bowel wall; however, even with this transducer the edematous bowel wall, free fluid, and pneumatosis were still visible.

Our case highlights the progression of ischemic colitis clinically, as well as on POCUS. On initial presentation, the patient had non-specific findings of colitis on POCUS: free fluid and a dilated bowel wall. However, our clinical suspicion was high for ischemic colitis. On the second ED visit, the patient presented with worsening symptoms of abdominal pain and diarrhea. Her second POCUS revealed signs of worsening disease, including significant enhancement of the pericolic fat, increasing amounts of free intra-peritoneal fluid, and evidence of bowel pneumatosis. Previous reports have shown that pneumatosis intestinalis was associated with severe ischemic colitis (e.g., requiring surgical intervention) 7. Our case echoes what previous studies have found and supports the use of ultrasound  in patients with suspected colitis. Our case also suggests not only the utility of POCUS in diagnosing colitis but also in predicting the severity of disease.

## Conclusion

Vague symptomatology and variable physical findings make ischemic colitis a challenging diagnosis for emergency physicians. POCUS has shown significant utility in diagnosing a variety of conditions of the bowel, including diverticulitis, colitis, and bowel obstruction 12, 13, 14, 15. In this case, abdominal POCUS was essential in making the early diagnosis of severe colitis, and prompted early initiation of antibiotics, CT imaging, and surgical consultation. Large-scale studies are needed to further evaluate the role of POCUS in diagnosing ischemic colitis. 

## Disclosure

AC is a consultant for Phillips Healthcare. All other authors report no relevant disclosures.

## Patient Consent

Informed consent was obtained from the patient prior to study inclusion. 
